# Enhancing the Performance of a Large Aperture Ultrasound System (LAUS): A Combined Approach of Simulation and Measurement for Transmitter–Receiver Optimization

**DOI:** 10.3390/s24010100

**Published:** 2023-12-24

**Authors:** Prathik Prabhakara, Vera Lay, Frank Mielentz, Ernst Niederleithinger, Matthias Behrens

**Affiliations:** Bundesanstalt für Materialforschung und -Prüfung (BAM), 12205 Berlin, Germany; prathik.prabhakara@bam.de (P.P.); vera.lay@bam.de (V.L.); frank.mielentz@bam.de (F.M.); matthias.behrens@bam.de (M.B.)

**Keywords:** engineered barrier system, monitoring, phased array technique, ultrasonic testing, non-destructive testing in civil engineering, geophysics, seismic migration, ultrasonic simulation, image reconstruction

## Abstract

The Large Aperture Ultrasound System (LAUS) developed at BAM is known for its ability to penetrate thick objects, especially concrete structures commonly used in nuclear waste storage and other applications in civil engineering. Although the current system effectively penetrates up to ~9 m, further optimization is imperative to enhance the safety and integrity of disposal structures for radioactive or toxic waste. This study focuses on enhancing the system’s efficiency by optimizing the transducer spacing, ensuring that resolution is not compromised. An array of twelve horizontal shear wave transducers was used to find a balance between penetration depth and resolution. Systematic adjustments of the spacing between transmitter and receiver units were undertaken based on target depth ranges of known reflectors at depth ranges from 5 m to 10 m. The trade-offs between resolution and artifact generation were meticulously assessed. This comprehensive study employs a dual approach using both simulations and measurements to investigate the performance of transducer units spaced at 10 cm, 20 cm, 30 cm, and 40 cm. We found that for depths up to 5 m, a spacing of 10 cm for LAUS transducer units provided the best resolution as confirmed by both simulations and measurements. This optimal distance is particularly effective in achieving clear reflections and a satisfactory signal-to-noise ratio (SNR) in imaging scenarios with materials such as thick concrete structures. However, when targeting depths greater than 10 m, we recommend increasing the distance between the transducers to 20 cm. This increased spacing improves the SNR in comparison to other spacings, as seen in the simulation of a 10 m deep backwall. Our results emphasize the critical role of transducer spacing in achieving the desired SNR and resolution, especially in the context of depth imaging requirements for LAUS applications. In addition to the transducer spacing, different distances between individual sets of measurement positions were tested. Overall, keeping the minimal possible distance between measurement position offsets provides the best imaging results at greater depths. The proposed optimizations for the LAUS in this study are primarily relevant to applications on massive nuclear structures for nuclear waste management. This research highlights the need for better LAUS efficiency in applications such as sealing structures, laying the foundation for future technological advances in this field.

## 1. Introduction

The ultrasonic pulse-echo method is of pivotal importance in the field of civil engineering, especially for the assessment of concrete structures such as bridges, thick foundations, dams, and structures in the nuclear industry [1,2,3]. In the context of concrete structures, ultrasonic testing plays an essential role in detecting latent anomalies, locating and assessing the condition of tendons, determining material characteristics, and performing accurate depth assessments [4,5,6,7]. The practice of this method is guided by a set of general guidelines, ensuring consistency and reliability in its application across various contexts [8,9]. Over time, the state of the art in ultrasonic testing has evolved significantly, incorporating advanced technologies and methodologies to enhance the precision, efficiency, and comprehensiveness of assessments, further solidifying its role as a critical tool in the maintenance and inspection of concrete infrastructures [10]. In addition, the use of advanced embedded sensor technology combined with cutting-edge analysis algorithms has opened new avenues in areas such as structural health monitoring and compliance with structural robustness standards [11].

The inherent heterogeneity of concrete, manifested in elements such as embedded reinforcements, inhomogeneous aggregate distributions, and different matrix constituents, often leads to acoustic disturbances. The requirement to use an acoustical coupler on non-uniform surfaces sometimes limits the widespread application of ultrasonic evaluations in civil engineering. Nevertheless, recent advances, such as the introduction of DPC transducer systems and the transition to low-frequency transducers (with frequencies of 20–100 kHz), are gaining momentum in the field of non-destructive structural evaluation [6,12,13].

In Germany, a stringent approach to radioactive waste disposal has been adopted, ensuring that it is isolated from inhabited areas. To achieve this, radioactive waste is stored deep underground, using special geological formations that provide stability and longevity. BGE, the federal government-owned operator for radioactive waste management in Germany, is responsible for this effort together with the supervising agency BASE [14]. Among the critical components of this safety-focused strategy are the sealing structures designed to provide an additional layer of protection. Recognizing the inherent stability of salt as a geological medium, the BGE has initiated an extensive research program. Central to this program is the Morsleben deposit (“ERAM”), a former salt mine selected for its favorable conditions when Germany was still separated. At this mine, a prototype sealing structure has been developed that consists of a specially developed salt concrete. This material, selected for its compatibility with the environment and its durability, is undergoing thorough testing to ensure that it meets the demanding requirements for the enclosure of radioactive waste [14]. 

In response to the challenges in waste management, BAM has initiated a special internally funded project called “Seal Waste Safe” (SWS) [15]. This project aimed to make progress on safe materials, multi-sensory monitoring, and ultrasound as quality assurance. The project emphasizes expanding sensor capabilities and highlights the importance of ensuring structural integrity via non-destructive evaluation, with a focus on ultrasonic testing methods. Within the broad range of SWS objectives, a prominent goal was to optimize the performance of the Large Aperture Ultrasound System (LAUS) by fine-tuning its capabilities. This fine-tuning is aimed at achieving higher accuracy in locating flaws, built-in parts, and voids, especially when deeply embedded in salt concrete structures [16,17].

The Large Aperture Ultrasound System (LAUS) has become an important technology for the non-destructive evaluation of large concrete structures to the characterization of materials and localizes (deeper) defects, voids, and embedded parts [18]. The LAUS system consists of twelve probe units, each equipped with shear horizontal wave transducers, designed for both transmitting and receiving ultrasonic waves. This configuration allows for deep penetration into concrete structures, achieving depths of up to circa 9 m. The versatility of the system is demonstrated by the ability to position the individual ultrasonic transducers on both flat surfaces [12,17,18] and curved surfaces in cylindrical test objects. To further extend the system’s capabilities and expand its range of applications to thicker/heavily reinforced concrete structures, the optimization of the performance of the LAUS is crucial. Here, we focus on increasing the potential depth penetration particularly needed for nuclear sealing structures by increasing the distance between the transducer units [12,18].

Recent developments in the field of geophysics are highlighted in studies investigating advanced 3D seismic reflection techniques for subsurface mapping. The work of Spitzer et al. demonstrates the application of a new 3D seismic reflection data set in delineating the subsurface structure of the Swiss Rhine Valley using innovative approaches such as re-gridding and trace sharing in the common midpoint (CMP) domain [19]. This method significantly improved the signal-to-noise ratio and resolution, resulting in a detailed representation of the lithologic layers in the subsurface. Another study highlights the effectiveness of 3D seismic surveying in imaging the ultra-shallow subsurface, capable of detecting multiple reflectors at depths of less than 20 m [20]. Critical to these advances are techniques such as amplitude offset analysis (AVO) and refined processing strategies to reduce errors in data interpretation. Taken together, these studies demonstrate the evolving capabilities and benefits of non-destructive testing methods in geophysical exploration and highlight their critical role in accurate subsurface characterization, which is essential for in-depth geophysical analysis and engineering applications [21].

In LAUS applications for sealing structures, the units are arranged close together to maintain a constant distance of 10 cm between each unit. This configuration enables the ultrasound waves to penetrate up to 9.6 m into salt concrete [16,18]. However, there is a need to investigate whether, by increasing the distance between these units, we can further enhance the penetration depth without compromising the horizontal and vertical resolution. By optimizing the transmitter–receiver distance, it may be possible to achieve greater penetration depths while maintaining the desired resolution, thus expanding the capabilities of LAUS.

In this research, we aim to systematically investigate and optimize the distance between the transmitter and receiver units of LAUS. The maximum distance between units is usually determined using the spatial sampling theorem for correct image reconstruction [22,23,24]. The main focus of this study is on the application of the Large Aperture Ultrasound System (LAUS) in salt concrete structures, especially the sealing structures needed for nuclear waste repositories. These structures have no reinforcement to prevent possible corrosion but are more than 10 m thick, and thus are characterized by their great depth, usually with no reinforcement. Thus, we focus on general identification of deep (<5 m) reflectors. In future steps, the imaging of embedded reinforcement at great depths as can be found in bridges, dams, or nuclear power plants can be assessed. Given their unique nature and their specific use in nuclear waste disposal, the study of embedded reinforcement is less relevant for this assessment.

First, simulations are carried out to find optimal measurement geometries applied in real measurements in a second step. In these simulations, different distances between the transducers and the depth and size of the reference borehole are analyzed. In addition, a 5 m long layer between two material properties and a 10 m long backwall are simulated, which resembles the concrete–salt transition layer in sealing structures. For the measurement phase, a 5 m thick concrete slab is used to test different transducer spacings. The spacings employed range from units close together at 10 cm, increasing in increments of 10 cm up to a maximum of 40 cm. Data from both simulations and measurements are reconstructed using Kirchhoff migration. A quantitative analysis is then performed, comparing the signal-to-noise ratio (SNR) of the reconstructed images. This comprehensive approach, combining simulations with real measurements, is crucial for advancing LAUS capabilities in ultrasonic imaging and testing methods, especially in the context of deep reinforcement-free structures such as those used to seal radioactive or toxic waste repositories.

## 2. Methods

### 2.1. Large Aperture Ultrasound System (LAUS)

The Large Aperture Ultrasound System (LAUS) used in this study is based on BAM’s unique ultrasonic testing device [12]. The LAUS has proven efficient in ultrasonic pulse-echo inspections, achieving penetration depths of up to ~9 m, particularly in the unreinforced concrete of underground sealing structures. It is specifically designed for inspecting thicker concrete structures with limited access on one side.

**Figure 1:**
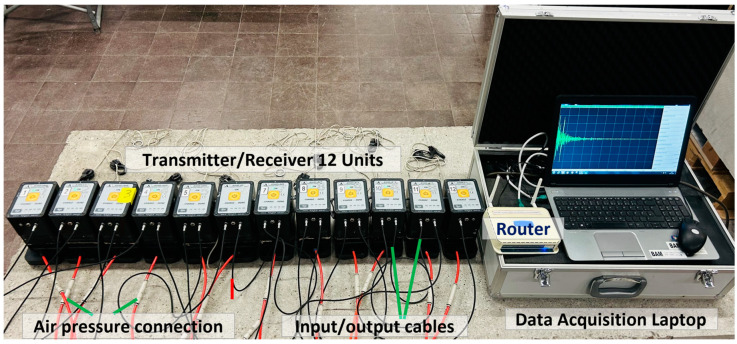
Large Aperture Ultrasound System (LAUS), 12 units placed equidistantly.

**Figure 2:**
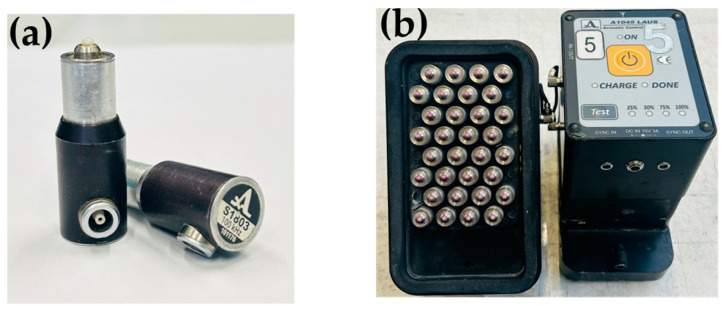
**(a**) Single DPC shear wave transducer 50 kHz center frequency [13], (**b**) single ultrasound unit consisting of 32 DPC transducers.

The LAUS system consists of a multistatic array of 12 identical probe units. These probe units can be flexibly placed on the concrete surface or used individually by spacing the transducers equally side by side as shown in Figure 1. Each probe unit is equipped with 32 horizontal dry point contact (DPC) shear wave transducers operating in the frequency range of 25–150 kHz (Figure 2a,b) [7,12,17]. The transducers are attached to the concrete surface with a vacuum housing with the aid of an external pressure system.

The LAUS system incorporates Dry Point Contact (DPC) transducers that are excited by electric pulses generated by the electronic module. The synchronization of ultrasonic signals is achieved using both radio channel and cable, ensuring a time lag of less than 1 μs. The acquired LAUS measurements are processed using the InterSAFT software version V1310, which is specifically designed for ultrasonic data analysis and uses advanced signal processing algorithms for Synthetic Aperture Focusing Technique (SAFT) reconstruction [4,5].

**Figure 3:**
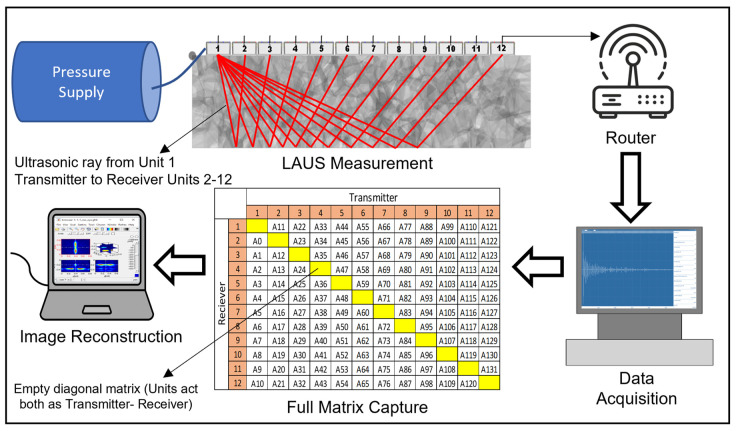
LAUS data acquisition and image reconstruction.

Figure 3 illustrates the data acquisition process of LAUS. The data collection is facilitated using a specialized extension program crafted for LAUS by Acoustic Control Systems, Ltd. (Saarbrücken, Germany), which is connected to the transducer units using a router. This program not only sets parameters tailored to various measurement applications, but also offers real-time tomographic visualization. During the acquisition, each transducer unit emits an ultrasound shear horizontal wave that is polarized perpendicularly [25]. These waves are captured by all other transducer units, but notably, the transmitting unit does not act as a receiver simultaneously (Full Matrix Capture in Figure 3). Upon completion, the system accumulates a total of 132 A-scans, which are saved in a raw file format. These raw data are then externally processed for signal processing and subsequently reconstructed using diverse imaging methods [26,27]. Presently, the SAFT method, with parameters pre-set tailored for LAUS in the InterSAFT software, is employed for data reconstruction [12,28].

### 2.2. Simulation

The CIVA software, developed by the French Alternative Energies and Atomic Energy Commission (CEA), is based on a finite element technique combined with a semi-analytical strategy for simulation and analysis in the fields of Non-Destructive Testing (NDT) and evaluation [29,30]. Within the ultrasonic testing module, there is a section dedicated to beam analysis, dealing with ultrasonic wave propagation characteristics. In addition, the inspection simulation module is tailored to simulate ray-based ultrasonic reflections that correspond to various reference defects and thus mimic real-world measurement scenarios [13,29,30].

**Figure 4:**
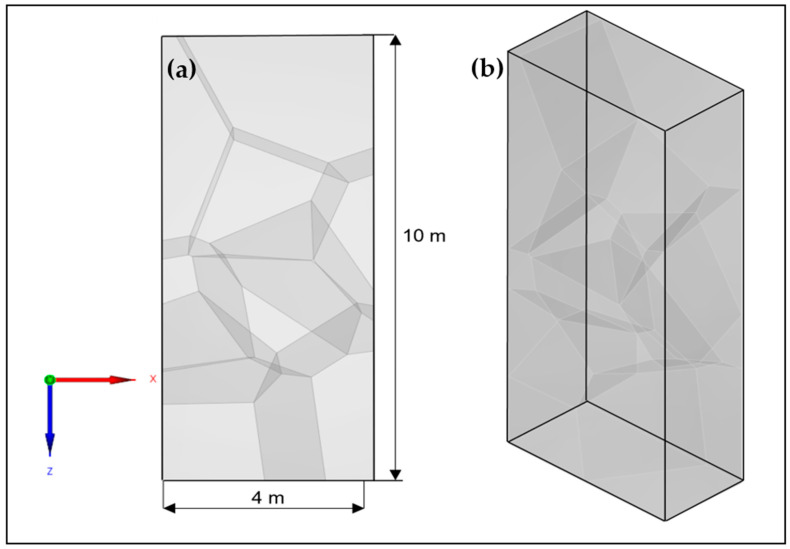
3D CAD model with integrated coarse grain structure (**a**) Front View; (**b**) orthogonal view.

For the 3D simulation, a model with dimensions of 4 m in length, 1 m width, and a 10 m thick plane surface is used (Figure 4). The material specified for this simulation is concrete, characterized by a shear wave velocity of 2700 m/s and a density of 2.6 g/cm^3^. To emulate the heterogeneous structure observed in real-world concrete, we incorporate a heterogeneous structure using a 3D Voronoi model. This model consists of 10 different cells, each with a random shear wave velocity variation of +/−5%, which is specified during parameter configuration. The cells within this heterogeneous model are assumed to be larger, which is due to the changes in concrete properties during the setting process. It should be noted that this model does not consider meso- and microscale inhomogeneities such as pores and aggregates, as the computational time required would be too high. Figure 4 shows the CAD model, highlighting the integrated coarse grain framework.

**Figure 5:**
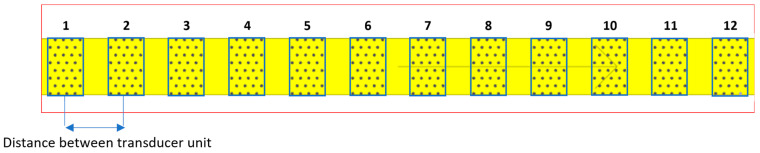
LAUS array for the simulation.

Within CIVA, the LAUS array is designed so that each unit comprises 32 shear horizontal (SH) waves. These SH waves are polarized perpendicular to the testing plane and are synchronized within the array unit, which comprises 12 transducer units as shown in Figure 5. For our study, we varied the spacing between units, using distances of 10 cm, 20 cm, 30 cm, and 40 cm to create distinct simulation configurations. The signal parameters were established based on specifications provided by the transducer manufacturer. In the simulations, the signal was characterized by key parameters: a central frequency of 25 kHz, a 50% bandwidth at a 3 dB drop, 8192 samples, and a 1 MHz sampling frequency. In terms of array operations, each unit sends out ultrasonic waves that are detected by the other units equivalently to real measurements.

We designed our simulation model and properties based on a specimen with a horizontal reference borehole (parallel to the surface on which the instrument is placed) used to assess borehole measurements [13]. For the purposes of our research, we designed four distinct simulation scenarios, described as follows:

- Retaining a consistent spacing between units while varying the diameter of the borehole to assess the impact of hole size on the results.
- Maintaining both the inter-unit spacing and the reference borehole’s size constant but adjusting the borehole depth to determine its influence on the readings.
- Keeping the depth and diameter of the borehole unchanged, the spacing between each unit was altered, ranging from 10 cm to 40 cm. This was performed to observe the effects of varying unit distances on the simulation output.
- Employing a model that incorporated two different material layers and examining the results for different unit spacings.

**Figure 6:**
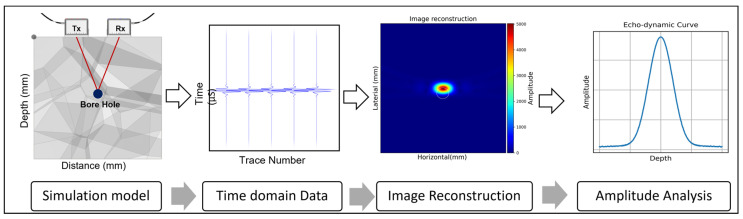
Process flow of simulation.

At the end of the simulation, reflection data were generated in the time domain, which were represented as A-scan signals. These signals underwent reconstruction using the SAFT (Synthetic Aperture Focusing Technique) within the designated simulation software. The analysis that followed concentrated on the distribution of amplitude in the reconstructed images. By comparing the results from various simulation setups, we aimed to identify trends and gain deeper insights. Figure 6 shows the process flow of the simulation.

### 2.3. Measurement and Analysis

**Figure 7:**
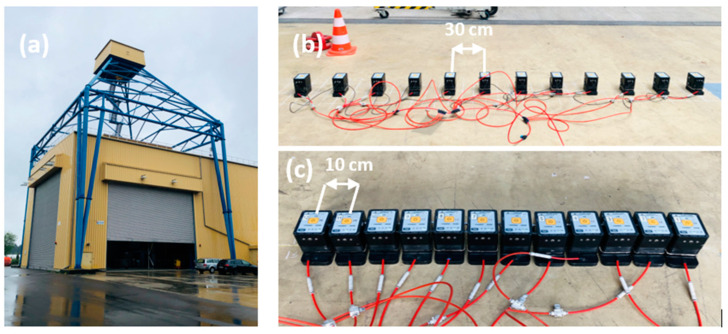
(**a**) Drop tower at BAM test site, (**b**) LAUS measurement with 10 cm spacing between transducer units, and (**c**) 30 cm spacing between transducer units.

**Figure 8:**
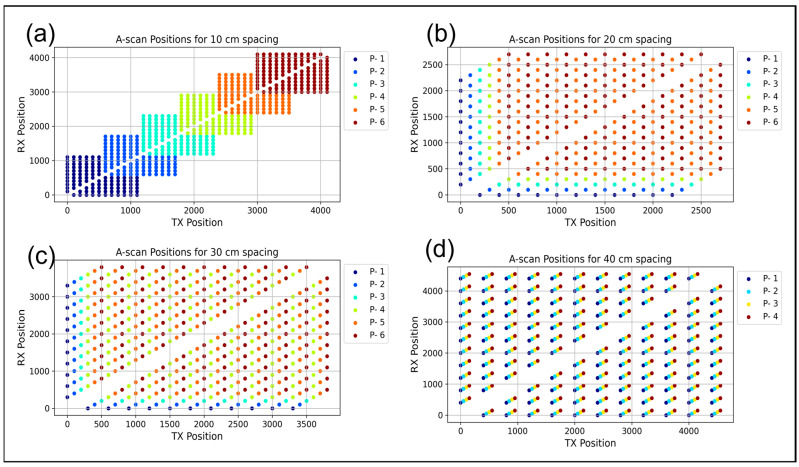
A-scan transmitter and receiver positions of the measurements on the concrete foundation. (**a**) 10 cm spacing, (**b**) 20 cm spacing, (**c**) 30 cm spacing, and (**d**) 40 cm spacing between each unit.

In alignment with the simulation framework, the measurements were carried out using a center frequency of 25 kHz on the BAM test site on the solid concrete foundation of the large drop tower (see Figure 7). This 5 m thick concrete foundation contains comprehensive reinforcement. The spacings between transducers were adjusted for the measurements, with spacings set at intervals of 10 cm, 20 cm, 30 cm, and 40 cm. The data collection methodology is explained in Section 2.1. The offsets for the measurement positions corresponding to the different distances are shown in Table 1. Also, the A-scan positions for the different transmitters and receivers are shown in Figure 7. In practical terms, when adopting a 10 cm spacing, the entire array was shifted by half its size to mimic the most on-site inspection tasks. For spacings of 20 cm, 30 cm, and 40 cm, the array offsets were 10 cm, 10 cm, and 5 cm, respectively, as summarized in Table 1 and Figure 8. Following data acquisition, the basic Kirchhoff migration method was used to analyze the measured data, focusing on the A-scans of selected positions for quantitative evaluation.

### 2.4. Data Pre-Processing and Image Reconstruction

**Figure 9:**
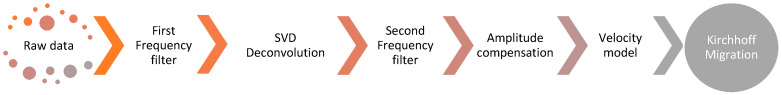
Data pre-processing steps and image reconstruction.

Raw ultrasound recordings often contain electronic and acoustic noise. To extract insightful detail from these data, careful processing and refinement is critical. Using scripts developed in Python, the data is pre-processed in several steps, as illustrated in Figure 9, and described in more detail in the following.

1. Frequency filtering (Bandpass filter): Ultrasonic waves generally operate within specific frequency bands. By limiting frequencies first from 5 kHz to 70 kHz and then to a narrower range of 10 kHz to 40 kHz, we can remove noise and focus on the most important frequency components [28,31,32].
2. Singular Value Decomposition (SVD) deconvolution: SVD is a powerful tool in linear algebra and signal processing that is used for various purposes including noise reduction, data compression, and system identification. In the context of seismic data processing, SVD can be employed to enhance the signal-to-noise ratio and to deconvolve the seismic signal. SVD is a factorization of a real or complex matrix. For a given matrix *A* of dimensions *m* × *n*, SVD decomposes *A* into three other matrices [28,32,33,34].
  (1) $$A=U\Sigma V^{*}$$
  where *U* is an *m* × *m* unitary matrix, indicating the left singular vectors of A. Σ is an *m* × *n* diagonal matrix with non-negative real numbers (singular values) on the diagonal, and V^*^ is the conjugate transpose of an *n* × *n* unitary matrix, representing the right singular vectors of A.

The singular values in Σ represent the strength of different components in the data, with larger values corresponding to the signal and smaller values associated with noise. In the context of seismic data processing, the matrix A represents seismic data, with rows corresponding to different traces and columns corresponding to time samples. To enhance the signal and reduce noise, a thresholding technique is applied to the singular values:

(2) $$S_{i}=\{\begin{array}{c} S_{i}\;if\;S_{i}\;\geq threshold.max\;\left(\Sigma \right)\\ 0\;if\;S_{i}\;\geq threshold.max\;\left(\Sigma \right) \end{array}$$

where threshold is a user-defined parameter between 0 and 1. This operation sets the small singular values (presumably corresponding to noise) to zero, while keeping the larger singular values (presumably corresponding to signal).

After thresholding the singular values, the matrix is reconstructed:

(3) $$A^{\prime }=U\Sigma^{\prime }V^{*}$$

where $\Sigma^{\prime }$ contains the threshold singular values.

This operation enhances the signal-to-noise ratio in the seismic data. However, the choice of the threshold is critical; too low a threshold might not sufficiently reduce the noise, while too high a threshold might remove essential parts of the signal. The optimal threshold often depends on the specific characteristics of the data and the desired outcome of the processing.

3. Signal amplitude compensation: As ultrasonic waves propagate through a medium, their energy decreases due to factors such as absorption, scattering, and mode conversion. This attenuation can vary depending on the properties of the medium and the distance the wave has travelled. Compensation techniques are essential for maintaining the clarity and consistency of received signals, especially when analyzing reflections from different depths.

Automatic Gain Control (AGC) is a method that adjusts the amplitude of received signals to a predefined level and ensures that they remain within a desired range and are comparable at varying recording times. This technique is particularly useful in real-time applications where the amplitude of the input signal may fluctuate dynamically and to enhance a weaker signal [31,35].

Time Varying Gain (TVG) is another approach in which the gain applied to the received signal is adjusted as a function of time. Since the time delay in receiving an echo in ultrasound is proportional to the depth from which it was reflected, TVG compensates for the loss of energy from signals coming from greater depths. This ensures that reflections from all depths are displayed with uniform intensity, allowing better interpretation of subsurface structures [31,35].

The use of these compensation techniques in ultrasonic testing ensures that the received signals are always clear and interpretable, regardless of their depth of origin. This is critical for applications such as defect detection, material characterization, and medical imaging, which enhance the later weaker reflection for correct analysis.

4. Kirchhoff migration: Reconstruction is an important technique in ultrasound imaging for converting reflected ultrasound signals from the time domain and assigning these reflections precisely to the corresponding physical locations [2,5]. Among the numerous reconstruction methods available, the Kirchhoff technique is characterized by the use of Two-Way Time (TWT) isochrones or can be seen as a diffraction summation based on the principle of superposition and Huygens’s principle [27,35,36,37,38,39]. Underlying Kirchhoff migration is the notion that each subsurface point interacts with multiple near-surface observation points. Conversely, the recorded signal of each of these surface receiver points is influenced by numerous visual points in the subsurface or concrete structure. According to [31,38,40], Kirchhoff migration in a 2D space can be represented mathematically as:
  (4) $$I\left(x,\;z\right)=\sum_{t_{x},r_{x}\in positions}A(t_{x},\;r_{x},\;T\left(x,\;z,\;t_{x},\;r_{x}\right).\;W(x,\;z,\;t_{x},\;r_{x}))$$
  where I(x, z) denotes the intensity of the migrated image *I* at specified spatial coordinates, with *x* representing the horizontal and *z* the vertical axis;$\;A\left(t_{x},\;r_{x},\;T\right)$ is the recorded seismic or ultrasonic signal amplitude for a source at $\;t_{x}$, a receiver at $\;r_{x}$, and travel time *T*; $T\left(x,\;z,\;t_{x},\;r_{x}\right)$ is the travel time from the source to the image point and back to the receiver, calculated based on the distances and the velocity of the medium; and $W(x,\;z,\;t_{x},\;r_{x}$) is the weighting function calculated based on the obliquity factor cos2θ [41,42] where *θ* represents the angle of incidence of the wave front with respect to the normal of the reflector. The intensity of reflections in ultrasound imaging depends on the angle of incidence. Perpendicular incidences (i.e., *θ =* 0⁰) give the maximum reflection intensity. When the angle deviates from this perpendicular orientation, the reflection intensity becomes weaker, so a compensation factor is required to ensure an accurate representation in the resulting image concerning reflection intensity.

In our study, we utilized the Kirchhoff migration method, implemented in the Python programming language, to accommodate various pre-processing steps that are unavailable using standard tools. For the simulation aspect of our study, the conventional tool Synthetic Aperture Focusing Technique (SAFT) was used, which is integrated into the CIVA software. The SAFT algorithm is based on the same principles as Kirchhoff migration, so reconstruction results are comparable.

## 3. Results and Discussion

### 3.1. Simulation

In this section, we present the results of our simulation based on the model described in Section 2.2. We employed a heterogeneous Voronoi model with 10 cells, each having a random velocity within +/−5% of the shear wave velocity of concrete, which is 2700 m/s. On this basis, we summarize the four scenarios in 3.1.1 Varying the Depth of the Borehole, 3.1.2 Varying Borehole Size, 3.1.3 Variation in Transmitter and Receiver Spacing, and 3.1.4 Investigation of a Two-Layer Material Model with Varied Unit Spacings: 

#### 3.1.1. Varying the Depth of the Borehole

**Figure 10:**
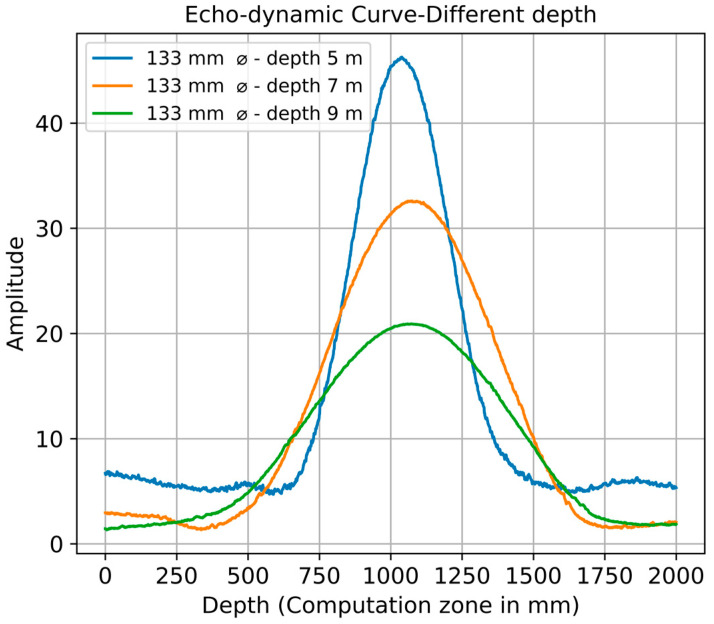
Echo-dynamic curve of variation in the borehole depth.

In this scenario, the dimensions of the borehole and the distance between the transmitter and receiver remain constant. Figure 10 shows the echo-dynamic curve along the x-axis of the reconstructed data for boreholes simulated at depths of 5 m, 7 m, and 9 m. With increasing depth of the borehole, there is a consistent and gradual decrease in signal amplitude. To obtain accurate numerical values, maximum amplitudes of 46, 33, and 21 were recorded for the respective depths. As ultrasonic waves propagate through a medium, including borehole boundaries, their interaction with the surrounding environment affects the recorded signal via attenuation, scattering, and energy dissipation. As longer travel paths increase these effects, lower amplitudes are observed for deeper boreholes. The half width of the echo-dynamic curve increases with the depth of the borehole, thus indicating a lower resolution. 

#### 3.1.2. Varying Borehole Size

**Figure 11:**
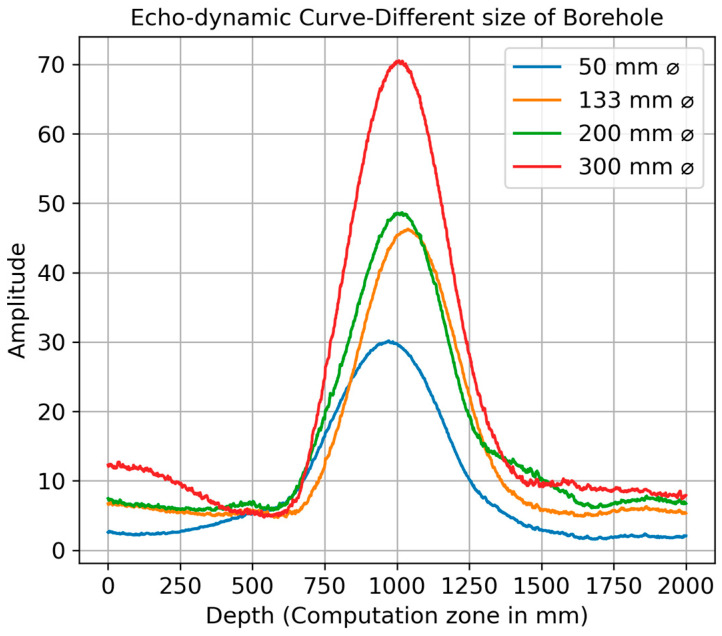
Echo-dynamic curve of different sizes of borehole.

In this specific simulation configuration, a constant spacing of 10 cm between the transmitter and receiver and a constant depth of the borehole was maintained. The only systematic changes were made to the diameter of the borehole. Figure 11 illustrates the echo-dynamic curve, effectively demonstrating the observed variations when analyzing different borehole sizes. The data from the echo-dynamic calculations show a clear and consistent trend: as the borehole diameter increases, there is a corresponding increase in the amplitude of the reflected signals. This trend can be seen in the maximum amplitudes recorded for different borehole sizes. For the smallest borehole diameter of 50 mm, the maximum amplitude is measured at 30.18. This amplitude increases with increasing borehole diameter. This amplitude increases with increasing borehole size, with a maximum amplitude of 46.28 measured for the 133 mm borehole, followed by 48.66 for the 200 mm borehole, and the highest amplitude of 70.56 for the 300 mm borehole. This indicates a direct correlation between the borehole diameter and the intensity of the reflected signals. Larger boreholes appear to favor stronger signal reflections, as shown by the progressively higher maximum amplitude values. This could be due to factors such as the increased surface area in larger boreholes, which could influence the reflection and absorption properties of the borehole walls, resulting in stronger signal reflections.

#### 3.1.3. Variation in Transmitter and Receiver Spacing

**Figure 12:**
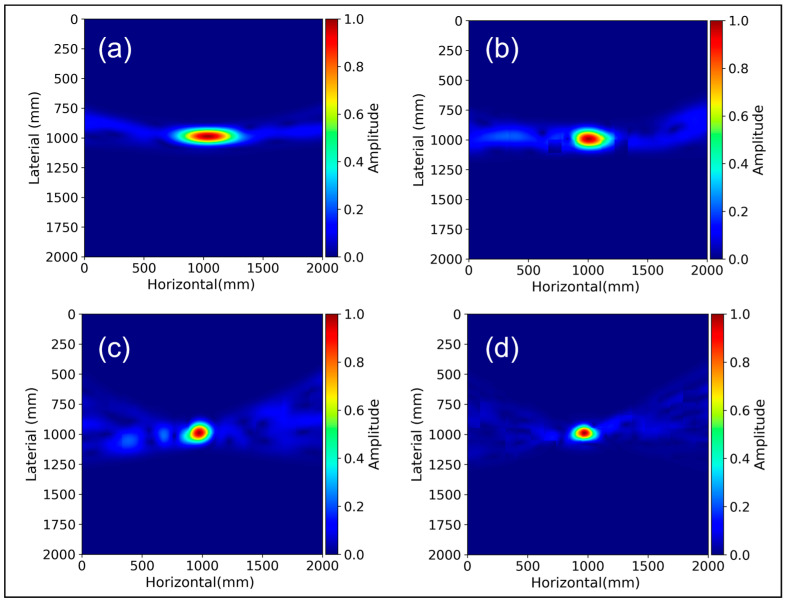
SAFT image reconstruction for borehole diameter of 133 mm within region of interest: (**a**) 10 cm spacing, (**b**) 20 cm spacing, (**c**) 30 cm spacing, and (**d**) 40 cm spacing.

To investigate the impact of different spacing configurations between transmitters and receivers, we first considered a borehole with a diameter of 133 mm placed at a depth of 5 m. We conducted separate simulations for transducer spacings of 10 cm, 20 cm, 30 cm, and 40 cm utilizing the LAUS settings. Figure 12 presents SAFT image reconstruction of simulated A-scan data for the various spacing configurations.

**Figure 13:**
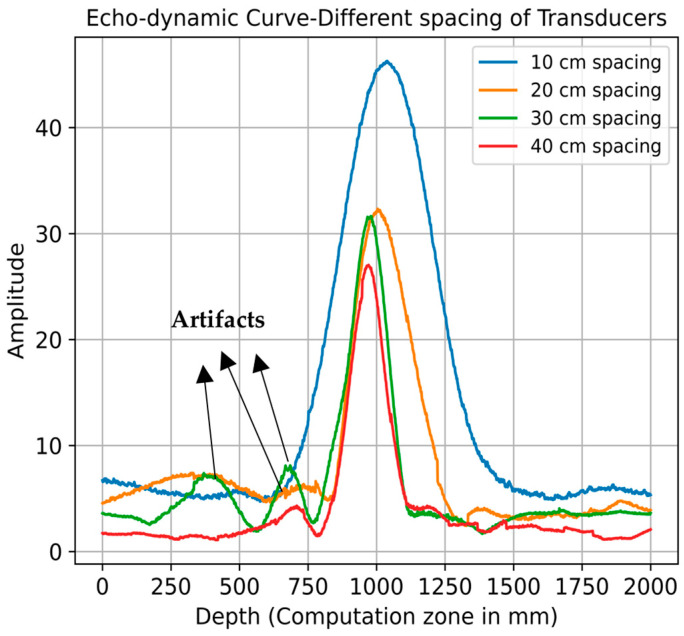
Echo-dynamic curve for different spacings of LAUS units, borehole diameter 133 mm.

Figure 13 provides a comparative analysis of echo-dynamic curves across various transducer configurations, extracting peak values from 2D image reconstructions. As transducer spacing widens, a noticeable reduction in the amplitude of reconstructed signals is observed, as illustrated in Figure 12d and Figure 13. This phenomenon can be attributed to the elongated propagation paths between the transmitter and receiver, leading to diminished signal strength.

The signal-to-noise ratio (SNR) of the echo-dynamic analysis (Figure 13) was determined to evaluate the resolution for different spacings. The noise window for this calculation was selected explicitly between 0 and 750 mm and from 1300 to 2000 mm depth. The results showed that the highest SNR of 17.81 dB was achieved at a distance of 10 cm, indicating the clearest signal. However, the SNR fluctuated with increasing distance: it dropped to 15.21 dB at 20 cm, further to 12.53 dB at 30 cm, and then increased slightly to 13.63 dB at 40 cm. This pattern suggests that although the clarity of the signal generally decreases with increasing distance, the relationship is not strictly linear, as shown by the unexpectedly lower SNR at 30 cm compared to 40 cm. 

In addition to these results, Figure 13 illustrates the changes in the echo-dynamic curve with increasing distance between the transducers. At larger distances, the resolution decreases noticeably, as evidenced by the decreasing amplitude of the echo-dynamic curve. At the same time, the occurrence of artifacts increases significantly, which are probably due to diffraction effects or interference patterns and become clearer as the distance between the transducers increases. This observation is consistent with the principles of wave propagation and illustrates the complicated relationship between resolution, signal strength, and artifact prevalence in echo-dynamic imaging. 

#### 3.1.4. Investigation of a Two-Layer Material Model with Varied Unit Spacings

**Figure 14:**
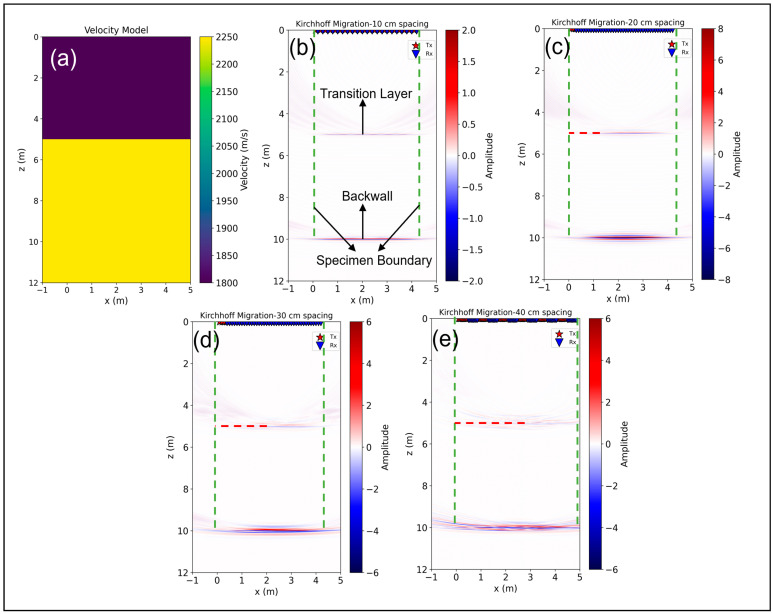
Kirchhoff migration of 2 different materials for the simulated data: (**a**) Velocity model of two layers, (**b**) 10 cm spacing, half array movement, (**c**) 20 cm spacing, 10 cm array movement, (**d**) 30 cm spacing, 10 cm array movement, and (**e**) 40 cm spacing, 5 cm array movement.

The objective of this study is to evaluate the performance of a two-layer material model with different distances between transmitter and receiver in an ultrasonic testing scenario. The model consists of two different homogeneous material layers: The first layer extends from 0 to 5 m with a shear velocity of 1800 m/s, while the second layer extends from 5 to 10 m with a shear velocity of 2250 m/s. These velocity values were derived from typical salt concrete found at the BGE test site and characterized by varying salt concentrations. The simulation was conducted with the settings of LAUS, as described in Section 2.2 above, utilizing an absorbing side boundary. The simulated data are then processed using Kirchhoff migration considering the input velocity model for ray-based calculations. The reconstruction results shown in Figure 14 show various results based on the different distances between the transducers. In Figure 14b, the 10 cm spacing effectively reconstructs the entire horizontal transition from salt to salt at a depth of 5 m and accurately locates the backwall at 10 m. In Figure 14c, the 20 cm spacing reveals high energy in both the backwall and the transition layer. It is shown by the red dashed line, which indicates a decreasing energy profile from 0 m to 1 m horizontally at 5 m depth. Nevertheless, the backwall shows a clear and strong energy characteristic at a depth of 10 m. 

As the distance increases to 30 cm in Figure 14d, the clarity of the transition layer decreases, but a significant increase in energy can be seen at 10 m depth. However, this increase is accompanied by increased ultrasonic scattering at a depth of 5 m. Finally, Figure 14e shows a less distinct transition layer and stronger scattering effects for a spacing distance of 40 cm. But this configuration results in more energy being detected in the backwall, illustrating the effects of different transducer distances on the reconstruction results.

Table 2 provides a comparative analysis of the signal-to-noise ratio (SNR) for different spacing and offset configurations between the scanning positions. It can be observed that the SNR for the transition layer degrades as the spacing between transducers increases from 10 cm to 40 cm with half an array offset configuration. However, an intriguing pattern is evident in the backwall echo at a 20 cm spacing where the SNR shows improvement. This uptick in SNR is likely related to factors inherent to the acoustic properties and interaction at this spacing. Subsequently, at spacings of 30 cm and 40 cm, a slight reduction in SNR is observed. Looking at the entire data set with decreasing offset, a consistent pattern emerges where the 10 cm spacing gives the highest SNR. In particular, the 20 cm spacing configuration shows an improved SNR for the backwall echo at 10 m.

**Figure 15:**
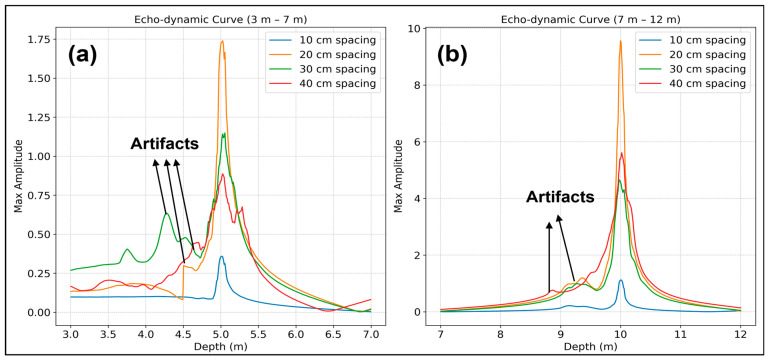
Echo-dynamic curve of maximum enveloped Kirchhoff migration: (**a**) Transition layer at 5 m, (**b**) backwall at 10 m.

Figure 15 illustrates the echo-dynamic curves for different spacings for the transition layer and the backwall for the full data set. Due to the lower offsets between scanning positions, the full data migration at 20 cm, 30 cm, and 40 cm spacing benefits from an increased number of A-scan stacking compared to the 10 cm spacing, resulting in a higher maximum amplitude observed in both the transition layer and the backwall. However, the plots also show artifacts that are more pronounced in the transition layer due to increased scattering, as well as a broadening of the curve width as the spacing is increased. In contrast, the amplitude distribution for the backwall is more favorable and shows only a minimal influence of artifacts. The 20 cm distance shows a higher penetration depth with the highest amplitude among the tested configurations.

The observed phenomenon indicates that the ultrasonic energy achieves deeper penetration into the material under test as the distance between the scanning positions increases. In particular, the configuration with 20 cm spacing proves to be optimal. It provides a balance by allowing greater penetration depth with acceptable resolution. 

### 3.2. Measurement

**Figure 16:**
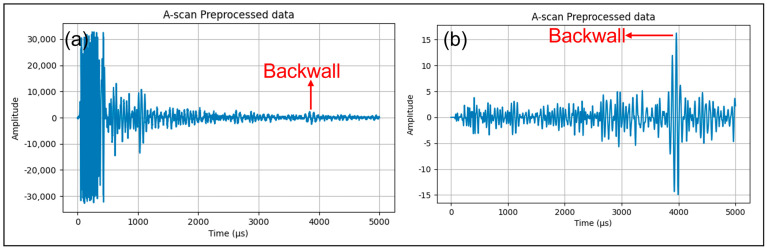
LAUS measurement: (**a**) A-scan raw data, (**b**) A-scan pre-processed data.

**Figure 17:**
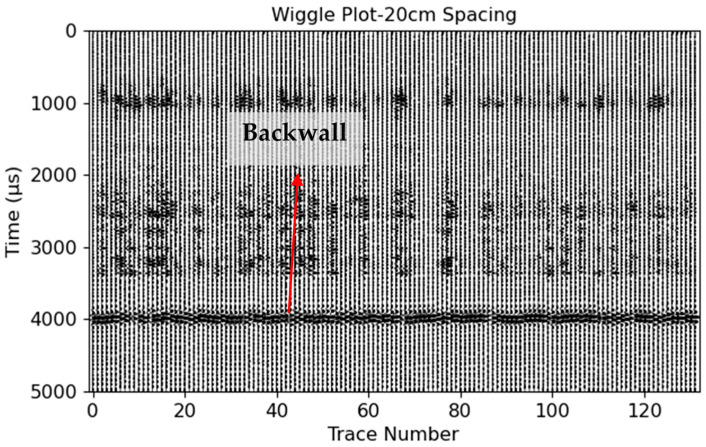
Wiggle plot for the 20 cm spacing of all 132 A-scans.

The assessment was carried out on a reinforced 5 m thick concrete foundation, as depicted in Figure 7, utilizing the LAUS configuration, which varied in terms of spacings and offsets as specified in Table 1. The methodology for data collection was consistent with the approach illustrated in Figure 3. Following this, the collected raw data were subjected to a series of pre-processing procedures, as outlined in Section 2.4. The specific parameters used for each procedure are detailed in Appendix A, Table A1. These parameters were carefully selected based on their ability to enhance the signal of the backwall reflection, ensuring the reliability of our results. It is crucial to note that the reproducibility of our results is dependent on these parameters. Figure 16b and Figure 17 display the A-scan and wiggle plot of the pre-processed data in the time domain, respectively, highlighting the backwall picked at the onset of the signal, which occurs at approximately 4000 microseconds. The Kirchhoff migration method was applied with the assumption of a constant velocity of 2700 m/s.

**Figure 18:**
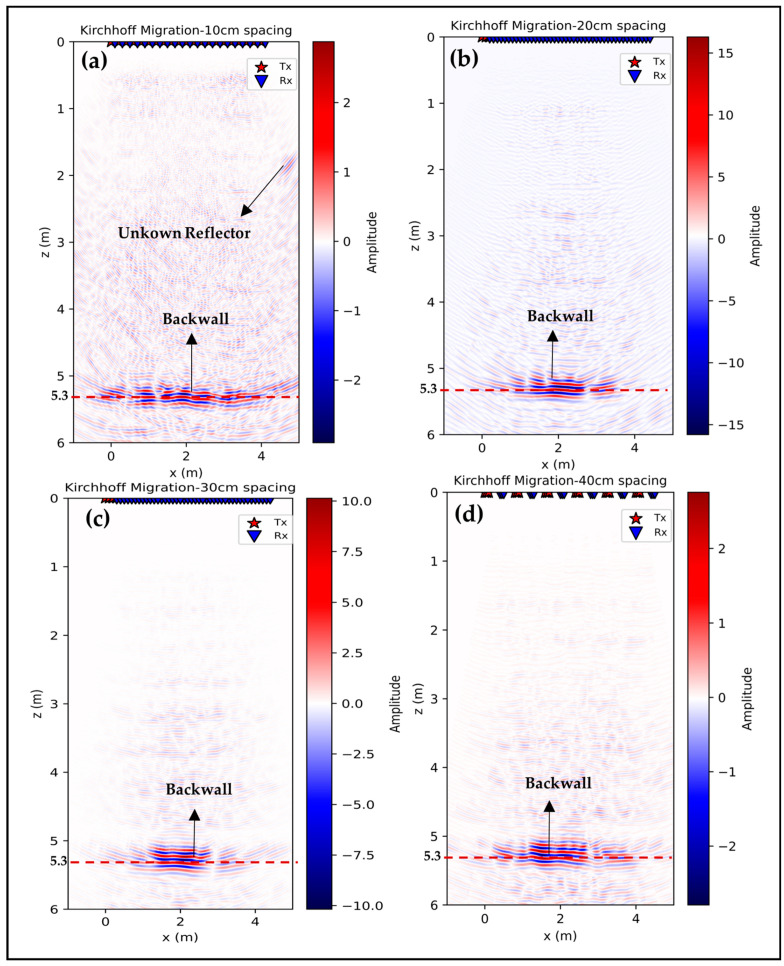
Kirchhoff migration of measurement data: (**a**) 10 cm spacing half array movement, (**b**) 20 cm spacing 10 cm whole array movement, (**c**) 30 cm spacing 10 cm array movement, and (**d**) 40 cm spacing 5 cm array movement.

The pre-processed measurement data were reconstructed using the Kirchhoff migration technique, as explained in Section 2.4, fourth section. The number of A-scans used for the reconstruction varied for different spacings, determined by the extent of the horizontal distance covered by the transducer positions. For the 10 cm spacing, 216 A-scans were used, for 20 cm 792, for 30 cm 432 and for 40 cm 108 A-scans. Figure 18 shows the reconstructed images for these different distances, which show a close correlation with the phenomena observed in our simulations, especially with the transition layer located 5 m below the subsurface. Figure 18a shows the reconstructed image obtained with a transducer spacing of 10 cm, with the measurement offset set to half the array size. This configuration allowed the location of the entire backwall at a depth of 5.3 m as well as the identification of an unknown reflector at x = 4.5 m, z = 1.9 m. The image is characterized by its clarity and sharpness which underlines the effectiveness of the chosen spatial arrangement for ultrasound imaging. In Figure 18b, the spacing of the transducers is adjusted to 20 cm, with the measurement offset shifting the array by 10 cm along the inspection line. Despite the altered array configuration compared to the 10 cm spacing, the backwall is still accurately localized at a depth of 5.3 m, demonstrating agreement with the results of the smaller spacing. Noticeably, an increase in amplitude can be observed as well as a slight scattering effect. These phenomena slightly mask the full visibility of the backwall. The increase in amplitude is likely due to the higher number of A-scans employed for reconstruction for this spacing. 

In Figure 18c,d, in which the distances between the transducers are increased to 30 cm and 40 cm, respectively, with corresponding measurement offsets of 10 cm and 5 cm, notable changes in image characteristics are observed. The reflection from the backwall becomes less distinct as the distance is increased. This effect may be explained by the altered propagation and interaction of the ultrasonic waves at greater distances. The wavefronts tend to cluster more in the center of the image, resulting in a higher intensity in this area. In addition, the phenomenon of constructive interference, which results from the superposition of the wavefronts in phase, causes an increase in the overall amplitude. However, this increase in amplitude, combined with the increased scattering, can obscure the finer details of the image and reduce the sharpness and visibility of subtle structural features.

### 3.3. Data Analysis of Migration Data

**Figure 19:**
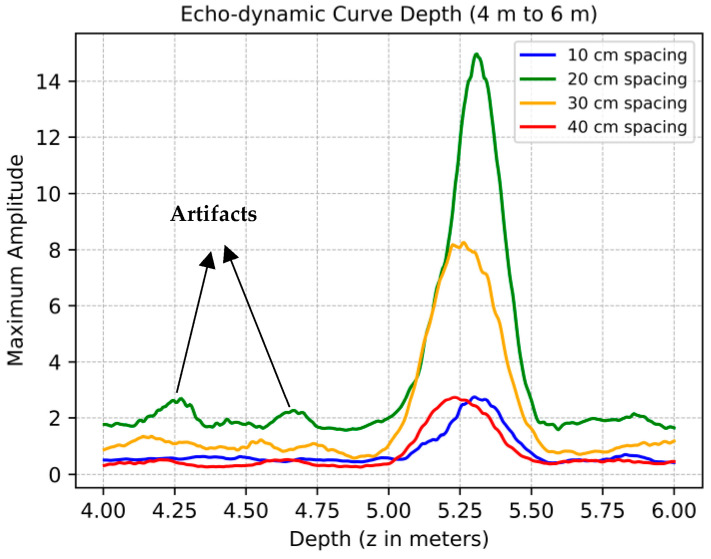
Echo-dynamic curve of maximum enveloped amplitude of migration data for the backwall.

In this section, the focus is on the reconstructed data obtained at varying distances, particularly examining the variations in mean amplitude along the horizontal axis. From the presented results, envelopes of the data are extracted to better pick the exact depth and compare results from various configurations. The echo-dynamic curves in Figure 19 illustrate the changes in the maximum amplitude of the envelope in relation to depth at the backwall reflection. Additionally, Table 3 presents the statistical distribution of migration data across different spacings. For each configuration, the selected depth of the backwall was determined based on the peak amplitude of the envelope in the range of 4 m to 6 m. This depth measurement is accompanied by a corresponding standard deviation of the amplitude at the backwall and the SNR. The SNR was calculated by taking the average signal amplitude at the backwall and dividing it by the average noise level measured from 0 to 4 m.

The reflection layer shows uniform peak depth values in all data sets, which are consistently in the range of 5.3 m. This shows a minimal variation compared to the actual backwall depth specified in the drawing. Among these, the data set with 20 cm spacing stands out, exhibiting a significantly enhanced peak amplitude. This amplification may stem from a denser reconstruction process that integrates a larger quantity of A-scans than its counterparts. The improved detection of subsurface features at this spacing is a possible explanation for this observation.

Comparative analysis reveals that the configurations with 20 cm and 30 cm spacings record higher peak amplitudes. This trend could be the result of more closely spaced measurement points and the smaller offset in measurement positions, which contrasts with the results from the more widely spaced 40 cm and large position offset for 10 cm configurations.

In terms of signal quality, quantified by the signal-to-noise ratio (SNR), all data sets perform well with values above 10 dB [4,43,44,45], ensuring a clear distinction between the signal and the background noise. In particular, the data set with a distance of 10 cm achieves the highest SNR, indicating better signal quality relative to noise, which is critical for generating clear and interpretable seismic images. In contrast, the data set with a spacing of 40 cm has the lowest SNR despite its lower standard deviation. This could be an indication of weaker signal strength or higher noise in this particular data set. On the other hand, the data set with 20 cm spacing has the highest standard deviation, indicating a higher degree of variability in the data. Conversely, the data sets with 10 cm spacing have a lower standard deviation, suggesting a more concentrated and uniform distribution of data points. In summary, this analysis section not only highlights the consistent presence of a reflective layer in all of the data sets, but also demonstrates the differences in signal quality and data variability associated with the different spacing. These findings are invaluable for interpreting ultrasonic data and understanding subsurface properties.

## 4. Discussion

### 4.1. Impact of Borehole Depth and Size

The simulation results demonstrate a clear trend of decreasing signal amplitude with increasing borehole depth, which is consistent with theoretical expectations due to increased signal attenuation over longer travel paths. This phenomenon was well illustrated in the echo-dynamic curves, where deeper boreholes also exhibited broader curves, indicative of lower resolution. On the other hand, increasing the borehole size resulted in an increase in the amplitude of the reflected signals, which could be attributed to less signal attenuation and scattering within larger boreholes. To evaluate the validity of these simulation results, it is essential to compare them with data from actual field tests with boreholes and embedded parts on test sites. The model in its current setup may not capture the full range of variables present in situ, suggesting that a more detailed and sophisticated approach could improve the predictive capabilities of the model. The insights gained from this investigation are of great importance for the further development of LAUS techniques in subsurface exploration. By integrating an improved simulation framework, our understanding of how acoustic signals behave in borehole scenarios could become more nuanced. It is imperative that future research strives for consistency between simulated predictions and empirical observations and pushes the boundaries of current simulation techniques to improve their reliability and applicability in real-world scenarios.

### 4.2. Transducer Spacing

The investigation into varying transducer spacings revealed a decrease in signal amplitude and resolution with increasing spacing. This is expected due to longer signal paths and increased chances of signal scattering and attenuation. The two-layer material model simulation highlighted how different spacing configurations could optimize the balance between penetration depth and resolution. Within the context of the material properties under consideration, a 20 cm transducer spacing emerged as a favorable trade-off. This observed trend was corroborated by measurement data acquired from a reinforced concrete foundation, which confirmed the simulation’s findings. The Kirchhoff migration results from the measurement data showed consistent localization of the backwall, although with increasing transducer spacing, scattering effects became more pronounced and the resolution decreased. Nevertheless, the time required for measurement increases as the offset between positions is reduced to gather more data for reconstruction and to locate reflectors more accurately. This involves balancing the depth of ultrasound penetration with the duration of the measurement. According to both simulation and actual measurements, a 10 cm spacing can provide clear resolution up to a depth of 5 m, even when the offset is relatively large.

### 4.3. Data Analysis Insights

The thorough analysis of migration data at different distances provided quantitative support to the observations made from the simulations and measurements. The variations in peak amplitude and signal-to-noise ratio across different spacings underscore the complex interplay between transducer spacing, signal quality, and resolution.

### 4.4. Comparative to Previous Studies

In geophysics, seismic imaging is closely related to ultrasonic imaging as both rely on elastic wave propagation. Seismic surveys require distinct planning strategies and need to deal with less accessible and more irregular surfaces due to vegetation or civil infrastructure than ultrasonic investigations. Thus, obtaining an optimal field layout is even more important and is specifically discussed for various applications [46]. Generally, a critical selection of sources and receivers rather than simply increasing their density is important for optimizing spatial configurations in seismic exploration, making them cost-effective and suitable for specific imaging purposes.

In ultrasonic imaging, basic source receiver geometries need less flexibility. Although there is no direct quantitative comparison for different transducer spacings, previous studies using LAUS in sealing structures with closely spaced units (approximately 10 cm apart) have shown high-resolution imaging of different reflectors at different depths. In particular, the Kirchhoff migration and reverse time migration results presented in the literature [18] show that a spacing of 10 cm is effective in locating reflectors up to a depth of 8 m. In another study using LAUS in magnesia shotcrete sealing structures with a spacing of 10 cm, reflectors up to a depth of 9.6 m were identified using SAFT reconstruction [17]. These field measurements support the results of our study, show the resolution possibilities of 10 cm spacing, and highlight the importance of advanced imaging techniques.

## 5. Conclusions

In this research, a comprehensive simulation and measurement study was conducted to investigate the impact of varying transducer spacings, borehole depths and sizes, and material models on ultrasonic data quality and resolution. The simulations provided valuable insights into how these parameters influenced the signal amplitude, resolution, and the presence of artifacts in the reconstructed images.

This study has significantly enhanced our understanding of the quality and resolution of ultrasonic signals, particularly in the context of backwall echo measurements in concrete structures, which are critical for assessing penetration depth. Via comprehensive simulations and field measurements, we have identified key factors that influence the results of ultrasonic testing using the Large Aperture Ultrasound System (LAUS). Our research shows that the distance between the transducers plays a crucial role in signal quality. We have found that a greater distance leads to a reduction in signal amplitude and resolution as signal paths become longer, and the likelihood of scattering and attenuation increases. However, we have also found an optimal transducer distance that provides a favorable balance between signal penetration depth and resolution. To investigate structures at depths greater than 10 m, we recommend 20 cm between the LAUS transducers. This recommendation is based on our findings that the reconstructed simulation data at this distance showed the highest signal-to-noise ratio (SNR) of 15.84 dB for a backwall simulated at a depth of 10 m. In addition, an SNR of 15.48 dB was determined when examining the reconstructed measurement data of a backwall at a depth of 5 m. This contrasts with an SNR of 16.40 dB observed at a smaller distance of 10 cm and an SNR of 13.22 dB at 40 cm. These results are significant in that they not only show the influence of transducer spacing on SNR, but also provide guidance for the optimal choice of transducer spacing when investigating deep subsurfaces. This finding is crucial for practical applications in ultrasonic testing, as the choice of transducer spacing can significantly affect the accuracy and reliability of results.

In addition to the distance considerations, our study also highlights the importance of data analysis in ultrasonic testing. The variations in peak amplitude and signal-to-noise ratio at different distances emphasize the complexity of interpreting ultrasonic signals. This highlights the need for stringent data analysis to accurately understand subsurface properties and the integrity of structures.

Looking to the future, this study opens possibilities for further research in this area. Future investigations could examine a wider range of testing conditions including flaws at greater depths or focus on embedded reinforcement. Additionally, advanced image reconstruction techniques will improve the accuracy and applicability of ultrasonic testing [47,48]. The refinement of data acquisition methods, such as the use of lower frequencies and higher sampling rates, will further improve the efficiency and effectiveness of these measurements.

In conclusion, this research provides a comprehensive understanding of the dynamics of ultrasonic testing, particularly in the context of backwall echo measurements in concrete structures. The knowledge gained is invaluable for refining inspection methods and improving the accuracy of subsurface exploration in various applications, including the assessment of critical infrastructure.

## Figures and Tables

**Table 1:** Measurement details of LAUS on concrete foundation.

Measurement		
Configuration No.	Spacing in cm	Position Offset in cm
1	10	60
2	20	10
3	30	10
4	40	5

**Table 2:** Signal-to-noise ratio (SNR) of Kirchhoff-migrated simulated data.

Depth Range	Spacing between Units (cm)	SNR (dB) Half Array Offset	SNR (dB) Full Data
Transition layer (5 m)	10	12.26	12.26
	20	5.29	9.20
	30	3.55	6.08
	40	2.99	4.07
Backwall (10 m)	10	7.29	7.29
	20	8.78	15.84
	30	7.74	12.14
	40	7.56	13.06

**Table 3:** Distribution statistics of migration data with different spacing.

Spacing	Peak Depth (m)	Peak Amplitude	Standard Deviation	SNR (dB)
10 cm	5.305	2.210	0.200	16.40
20 cm	5.304	11.470	0.883	15.48
30 cm	5.264	7.220	0.503	14.16
40 cm	5.259	2.382	0.160	13.22

## Data Availability

The data presented in this study are available on reasonable request from the corresponding author.

## Appendix A. Pre-Processing Parameters to Enhance Backwall Reflection Signal

**Table A1:** Parameters used for pre-processing of the raw data.

Parameter	Pre-Processing Step	Description	Value
Lowcut-1, Highcut-1	Bandpass Filter 1	cut-off frequencies for first bandpass filter	5 k Hz to 70 kHz
Lowcut-2, Highcut-2	Bandpass Filter 1	cut-off frequencies for second bandpass filter	10 k Hz to 40 kHz
Fs	Bandpass Filter 1, 2	Sampling frequency	1 M Hz
Window length before	Automatic Gain Control	Window length before reflection sample for AGC	1024 µs
Window length after	Automatic Gain Control	Window length after reflection sample for AGC	4096 µs
Start gain, End gain	Time-Varying Gain	Initial gain and Final gain for time-varying gain (TVG)	0 dB, 20 dB
Start time–end time	Time-Varying Gain	Start and end time in TVG	0–5000 µs
SVD threshold	SVD Deconvolution	Threshold for singular value decomposition (0 to 1 normalization)	0.5
Window size	SVD Deconvolution	Size of window for windowed SVD deconvolution	512 µs
